# Isolation and Characterizations of Histamine- and Tyramine-Producing Strains Isolated from Fermented Soybean Food: Soy Sauce and Soybean Paste

**DOI:** 10.3390/foods14142407

**Published:** 2025-07-08

**Authors:** Xiao Zhang, Sihao Li, Heng Liu, Anurak Wongta, Zhenlin Xu, Kai Zhou, Surat Hongsibsong

**Affiliations:** 1 Institute of Jiangxi Oil-Tea Camellia, School of Mechanical and Intelligent Manufacturing, Jiujiang University, Jiujiang 332005, China; zxjju2019@163.com (X.Z.); 18279483990@163.com (S.L.); 18779654795@163.com (H.L.); 2 School of Health Sciences Research, Research Institute for Health Sciences, Chiang Mai University, Chiang Mai 50200, Thailand; anurak.wongta@cmu.ac.th; 3 Guangdong Provincial Key Laboratory of Food Quality and Safety, Guangdong Laboratory of Lingnan Modern Agriculture, South China Agricultural University, Guangzhou 510642, China; jallent@163.com

**Keywords:** fermented soybean foods, histamine, tyramine, *Bacillus*

## Abstract

Histamine (HIM) and tyramine (TYM) are among the most toxic biogenic amines (BAs) commonly found in various fermented soybean foods, yet the crucial BAs-producing strains are ignored. This study discussed and compared the effectiveness of two methods based on medium pH screening and target gene amplification for identifying HIM- and TYM-producing strains from two fermented soybean foods. The crucial strains responsible for HIM and TYM formation were identified and then characterized. It was found that the strains forming large amounts of total BAs promoted a high pH at the final medium, but there was no correlation between TYM/HIM formation and the pH value among the isolates. Furthermore, a large portion of isolates that produce TYM/HIM cannot be amplified. The *hdc* and *tdc* genes utilized reported universal pairs of primers, resulting in false negative results. Following two rounds of screening, most TYM/HIM-producing strains were found to belong to *Bacillus*. *Bacillus cereus*-HT-31-2 and *Millerozyma farinosa*-HT-42-1 were identified as crucial producers of TYM and HIM in soy sauce during the fermentation stage, while *Proteus mirabilis*-T-24-2 was found to be the key producer of TYM in thua nao. Moreover, the simulated medium was found to be beneficial for the formation of TYM/HIM by *B. cereus*-HT-31-2 and *P. mirabilis*-T-24-2, but not for *M. farinosa*-HT-42-1. The formation of TYM/HIM was not synchronized under different conditions. This study provides insights into the key strain responsible for the formation of HIM and TYM in fermented soybean foods.

## 1. Introduction

Biogenic amines (BAs) are low-molecular-weight organic bases found in a wide variety of fermented beverages, soybean foods, sausages, and aquatic foods. There are eight major BAs in food, and they can be divided into three categories: heterocyclic amines, such as tryptamine and histamine (HIM), and these include spermine, spermidine, putrescine, and cadaverine, and aromatic amines, such as phenylethylamine and tyramine (TYM) [1,2]. While modest doses of BAs exhibit biological activities that can benefit human health, the excessive intake of BAs can easily cause allergies, especially in patients taking monoamine oxidase inhibitors (MAOIs) [3,4]. Among these BAs, HIM and TYM are the two most toxic, and they frequently lead to food safety incidents due to excessive intake within a short period of time. The consumption of foods high in HIM can cause toxicological reactions (neurological, gastrointestinal, circulatory, and respiratory symptoms; flushing, rashes, and urticaria), and this collection of symptoms is commonly referred to as “scombroid syndrome” [5]. Similarly, the consumption of foods high in TYM can cause tyramine interaction, a condition characterized by adverse headaches, migraines, neurological disorders, nausea, vomiting, respiratory disorders, and hypertension [2]. Notably, HIM and TYM are the major BAs found in high concentrations in some fermented soybean foods [6], and our previous research has highlighted the occurrence and risk of BAs in soy sauces and soybean pastes [7,8]. Exploring the production and accumulation of HIM and TYM in fermented soybean foods is crucial for reducing the risk of BAs poisoning.

BAs are typically formed through the decarboxylation of the corresponding amino acids. The accumulation of BAs in fermented foods is a highly intricate process that is dependent on several factors: the generation of amino acid precursors, the initial microbial composition (including the species and abundance of BAs-producing strains), amine oxidase activity, microbial interactions, and environmental conditions [9]. Strains containing decarboxylase genes have garnered increasing attention for due to their ability to convert amino acids into BAs [10]. Previous studies reported that some bacterial genera, such as *Bacillus*, *Clostridium*, *Lactobacillus*, *Pseudomonas*, *Enterobacteriaceae* and *Enterococci*, are able to generate BAs via the decarboxylation of amino acids [11]. However, the ability to produce BAs is not universal across species or strains within these genera; rather, it is a strain-specific trait. This specificity arises because BAs biosynthesis requires functional decarboxylase genes and associated metabolic pathways that are present only in particular strains [12,13]. Histidine can be converted to HIM through histidine decarboxylase (*hdc*), while tyrosine can be converted to TYM by tyrosine decarboxylase (*tdc*). Therefore, identifying strains carrying functional *hdc* or *tdc* genes capable of producing HIM or TYM will facilitate future strategies for reducing BAs levels.

Fermented soybean products are widely consumed because of their good taste and high nutritional value [14]. Two species, bacteria-fermented soybean products and mold-fermented soybean products, are commonly consumed around the world [15]. Bacteria-fermented soybean products include *natto* from Japan and thua nao from Thailand, and the starters or dominant microorganisms are usually *Bacillus* species, which contribute to the characteristic aroma and texture [16,17]. The representative mold-fermented soybean products are soy sauce, broad bean paste from China, and miso from Japan. The first step in mold-fermented soybean production is *koji*-making, which involves the inoculation of *Aspergillus* spp. in soybeans [18,19]. However, fermented soybean foods tend to be contaminated by high levels of BAs due to their abundant amino acids and BAs-producing microorganisms, as well as the fermentation methods used. High levels of HIM and TYM are found in soy sauce and thua nao. The maximum HIM content detected in soy sauce reached 477.79 mg/L [8], which may lead to HIM intolerance in susceptible people [20]. The highest TYM content was found in thua nao (548 mg/kg), which may pose a high risk for patients consuming monoamine oxidase inhibitors [10]. Recent studies have associated the genera *Bacillus*, *Lactobacillus* and *Enterococcus*, as well as *Enterobacteriaceae*, with the production of HIM and TYM in foods [21]. Given that most fermented soybean foods undergo fermentation (or potential contamination) by *Bacillus* species, this genus is also implicated in BAs production [22]. While metagenomic and/or metatranscriptomic analyses suggest that some microbial genera may be responsible for HIM and TYM synthesis [23,24,25], few studies have specifically identified the dominant BAs-producing strains responsible for HIM and TYM formation in these foods or characterized their properties. Consequently, researchers focused on the role of starter cultures in modulating HIM and TYM formation [26]. This partly from the challenge of directly inhibiting the growth of BAs-producing microorganisms, as pinpointing the specific culprits remains difficult. However, the open or semi-open fermentation environments typical of many soybean products allow environmental microbiota to colonize the fermentation matrix, potentially diminishing the dominance of inoculated starter cultures [22].

Undoubtedly, controlling HIM and TYM levels requires a precise understanding of the formation pathways and resulting strains. Robust techniques for screening BAs-producing microorganisms are still being developed and refined. Most current approaches rely on differential media containing pH indicators, exploiting the pH increase typically associated with BAs production. For instance, bromocresol purple-containing media are commonly used to screen fish products for HIM-producing bacteria [27]. However, there is no conclusive evidence that microbial decarboxylation leading to BAs formation necessarily causes detectable pH changes. This limitation may yield numerous false negatives or positives. Consequently, recent research has shifted towards advancing molecular techniques, particularly those targeting the *hdc* or *tdc* genes encoding HIM or TYM decarboxylases, to directly identify HIM- and TYM-producing microorganisms [28]. Notably, as BAs production is a strain-specific trait rather than a species-level characteristic [12], this implies disparities in the coding genes *hdc* or *tdc*, which impede the amplification of the target gene. The conventional quantification necessitates sophisticated instrumentation and involves time-consuming extraction and derivatization steps.

In this study, we examined representative fermented soybean products, soy sauce, and thua nao, and the HIM- and TYM-related microorganisms originating from these products were isolated and identified according to the change in pH, target gene amplification, and HPLC detection. The key HIM- and TYM-producing strains and their characteristics were explored and compared. This research provides insights into the critical strains generating HIM and TYM in fermented soybean products, enabling regulation of BAs accumulation in these foods.

## 2. Materials and Methods

### 2.1. Chemicals and Media

Tyrosine (>99.0%), histidine (>98.5%), TYM (98.0%), HIM (96%), 1,7-diaminoheptane (98.0%), dansyl chloride (D-Cl), bromocresol purple, tryptone, yeast extract powder, sodium chloride, 5′-phosphopyridoxal, glucose, sodium glutamate, sodium hydroxide, sodium bicarbonate, and ammonium acetate were purchased from Aladdin (Shanghai, China). Chromatography-grade acetonitrile, acetone, and diethyl ether (99.5%) were purchased from J.T. Baker (Wilmington, DE, USA). MRS, LB, and YPD media were purchased from Huangkai Biology Co. Ltd (Guangzhou, China).

DNA Ladder, DNA extraction kits, 2 × Taq PCR Mix kits, and a DNA product purification kit were purchased from TIANGEN Biotech Co., Ltd. (TIANGEN, Beijing, China). The primer was designed by Primer Premier 5 (Tsingke, Changsha, China) and synthesized by Tsingke Biotech Co., Ltd. (Tsingke, Changsha, China).

The amino acid medium consisted of 5 g/L tryptone, 5 g/L yeast powder, 5 g/L sodium chloride, 1 g/L calcium carbonate, 1 g/L histidine, 1 g/L tyrosine, and 0.05 g/L pyridoaldehyde phosphate, pH 5.5. The re-screening medium utilizes a *moromi* medium and a soybean medium. *Moromi* fermented on day 45 was thoroughly blended twice (*m*/*v*) with distilled water in a blender before filtration. The resulting supernatant was supplemented with 1 g/L of histidine and tyrosine, adjusted to a pH of 5.5 and 18% (*v*/*w*) salinity. The soybean medium was prepared from the powder obtained by pulverizing soybeans, which were extracted using ten times (*m*/*v*) water at 45 °C. The resulting supernatant was collected and supplemented with 1 g/L of histidine and tyrosine, adjusted to a pH of 5.5. *Moromi* and steamed soybeans were utilized as simulation fermentation medium to assess the ability of the screened isolates to produce HIM and TYM.

### 2.2. Strain Isolation

Soy sauce was provided by a factory from Zhongshan, China. The production processes for the *moromi* fermentation procedures were described in a previous report [29]. Mature thua nao was purchased in Chiang Mai Province, Thailand. The 30 days fermented *moromi* and mature thua nao were sampled and transported to a laboratory in ice boxes. Upon arrival, samples were homogenized aseptically, and serial 10-fold dilutions were prepared using sterile 0.85% saline solution. Aliquots (100 μL) of appropriate dilutions were spread onto three different media: MRS (for lactic acid bacteria), YPD (for yeasts), and LB (for general bacteria). Plates were incubated at 30 °C for 48–72 h. After incubation, colonies with distinct morphologies (e.g., size, shape, color, margin) were randomly selected from each medium to maximize the representation of the microbial diversity. For each morphological type, at least five colonies were picked. These colonies were purified by streaking three times consecutively on the same medium to ensure purity. Pure isolates were preserved in 30% (*v*/*v*) glycerol at −80 °C for further study.

### 2.3. Isolation and Preliminary Screening of BAs-Producing Strains

The pure colonies isolated from the fermented *moromi* were incubated in the liquid amino acid medium at 37 °C for 48 h, followed by centrifugation at 5000 rpm for 5 min and subsequent analysis. First, the supernatant pH was measured using a pH meter (Starter3100, OHAUS Co., Ltd., Parsippany, NJ, USA) to determine whether alkaline BAs were produced. The optical density at 600 nm (OD600) of the supernatant was measured using an absorbance microplate reader (Sunrise 300, Tecan Trading AG, Männedorf, Switzerland) to assess the growth status of biogenic amine-producing strains. Subsequently, polymerase chain reaction (PCR) was used to detect *hdc* and *tdc* genes associated with biogenic amine (BA) production. Finally, 1 mL of the supernatant was analyzed using HPLC for HIM and TYM.

### 2.4. Identification of Tyrosine and Histidine Decarboxylase Genes

The presence of the genes encoding *hdc* and *tdc* in DNA obtained from the isolates was determined using PCR amplification with the designed primer (Table 1). The PCR (T10S, Hangzhou Langji Scientific Instruments Co., Ltd, Hangzhou, China.) procedures were conducted as follows: an initial denaturation step at 94 °C for 5 min, 30 cycles, including 94 °C denaturation for 30 s, and primer binding at 56 °C for 30 s. The propagation step was performed at 72 °C for 1 min; after 30 cycles, the final propagation step was carried out at 72 °C for 5 min. PCR products were electrophoresed (HT-300 Beijing Saizhi Chuangye Technology Co., Ltd, Beijing, China.) in 1.5% agarose gel; then, the electrophoresis gel was stained with ethidium bromide 1 mg/mL and washed for 10 min before a gel image was obtained.

### 2.5. Secondary Screening and Isolate Identification

After 24 h of activation, the isolates were normalized at OD600 = 1 and inoculated. The isolates from the fermented *moromi* and matured thua nao were cultured in a *moromi* medium and a soybean medium, respectively, with an inoculation quantity of 1% (*v*/*w*). The isolates from *moromi* and thua nao underwent stationary culture at 30 °C for 7 days and 2 days, respectively, and then HIM and TYM were determined.

The genomic DNA of isolates with high levels of HIM and TYM accumulation was extracted using DNA extraction kits according to the manufacturer’s instructions. The universal primer pairs were used to amplify the bacteria 16S rRNA gene and fungal ITS rDNA gene targeting the internal transcribed spacer region. The 16S rRNA gene was amplified via PCR with the primers of 27F (5′-AGAGTTTGATCCTGGCTCAG-3′) and 1492R (5′-GGTTACCTTGTTACGACTT-3′). The amplification of the ITS region was achieved using PCR, and the applied primers were ITS1 (5′-TCCGTAGGTGAACCTGCGG-3′) and ITS4 (5′-TCCTCCGCTTATTGATATGC-3′). The PCR products were visualized using 1.0% agarose gel electrophoresis. PCR products were then purified and sequenced. The resulting sequences were analyzed using a BLAST search in the GenBank database (National Center for Biotechnology Information), and the phylogenetic tree construction was constructed using MEGA software (version 11.0).

### 2.6. Simulated Fermentation with the Screened Isolates

The HIM- and TYM-producing strains were cultured in NB medium, followed by centrifugation of the fermented broth at 3000 rpm for 10 min at 4 °C. The precipitation of the strains was resuspended in 0.85% saline. Subsequently, the prepared isolates were inoculated into fermented *moromi* and boiled soybeans at an inoculation quantity of 1% (*v*/*w*). After incubation for 7 days and 2 days, respectively, the contents of HIM and TYM in the samples were measured using HPLC.

### 2.7. Characteristics of HIM and TYM Formation by the Screened Isolates

The HIM- and TYM-forming abilities of the screened isolates were assessed under varying conditions, including different medium pHs, incubation temperatures, salinity levels, glucose, histidine, and tyrosine content in the medium, and light and oxygen conditions. Specifically, the temperatures tested included room temperature, 30 °C, and 37 °C; pH levels for the strains from *moromi* ranged from 4 to 6 (4, 4.5, 5, 5.5, and 6), while those for strains from thua nao ranged from 6 to 8 (6, 6.5, 7, 7.5, and 8); light control during culturing was achieved using tinfoil; the culture types include shaking and static conditions; salinity concentrations were set at 0, 1, 3, 6, 9, and 18% *w*/*v* for isolates from *moromi* and 0, 0.5, and 1% *w*/*v* for isolates from thua nao; concentrations of glucose were 0, 2, 5, 10, and 20 g/L; and concentrations of histidine and tyrosine were 0, 0.1, 0.5, and 1 g/L. The HIM and TYM contents of each sample were measured after the culturing.

### 2.8. Determination of BAs

Eight BAs, including TYM and HIM, were quantified using an Agilent 1260 HPLC-DAD, as described in our previous study [9]. Briefly, 1 mL of the liquid sample was added to 250 μL of 1,7-diaminoheptane (100 mg/L), 100 μL of 1 M NaOH, and 1 mL of saturated NaHCO_3_, and it then reacted with 1 mL of 10 mg/mL dansyl chloride at 60 °C for 15 min. The reactant was mixed with 100 μL of sodium glutamate and incubated at 60 °C for 15 min to remove the residual dansyl chloride. One milliliter of pure water was added into the tube and fully mixed, and the acetone was evaporated via N_2_ flow at 40 °C. After that, the sample was added to 0.5 g NaCl and then extracted twice with 3 mL diethyl ether. The complete extract was filtered through a 0.22 μm Millipore filter before we carried out the HPLC analysis.

The separation was carried out on an Aligent C18 column (250 × 4.6 mm, 5 µm) using a mobile phase composed of solution A (a mixture of 90% acetonitrile and 10% ammonium acetate solution (0.01 mol/L with 0.1% acetic acid)) and solution B (a mixture of 10% acetonitrile and 90% ammonium acetate solution (0.01 mol/L with 0.1% acetic acid)). The gradient program was as follows: 0–40 min, 40–15% B; 40–43 min, 15–0% B; 43–50 min, 0% B; 50.01–57 min, 40% B. The injection volume was set at 20 μL, the flow rate was 0.8 mL/min, the detector was set at 254 nm, and the column temperature was set at 35 °C.

### 2.9. Statistical Analysis

Data were analyzed using the Statistical Package for Social Sciences (SPSS), version 22, for Microsoft Windows. Analysis of variance was performed to determine the significant differences (*p* < 0.05) among samples. The diagrams were made using Origin Pro 2018. The results are presented as the average and standard deviation of triplicate samples.

## 3. Results and Discussion

### 3.1. Comparison of Screening Methods for HIM- and TYM-Producing Strains

**Figure 1:**
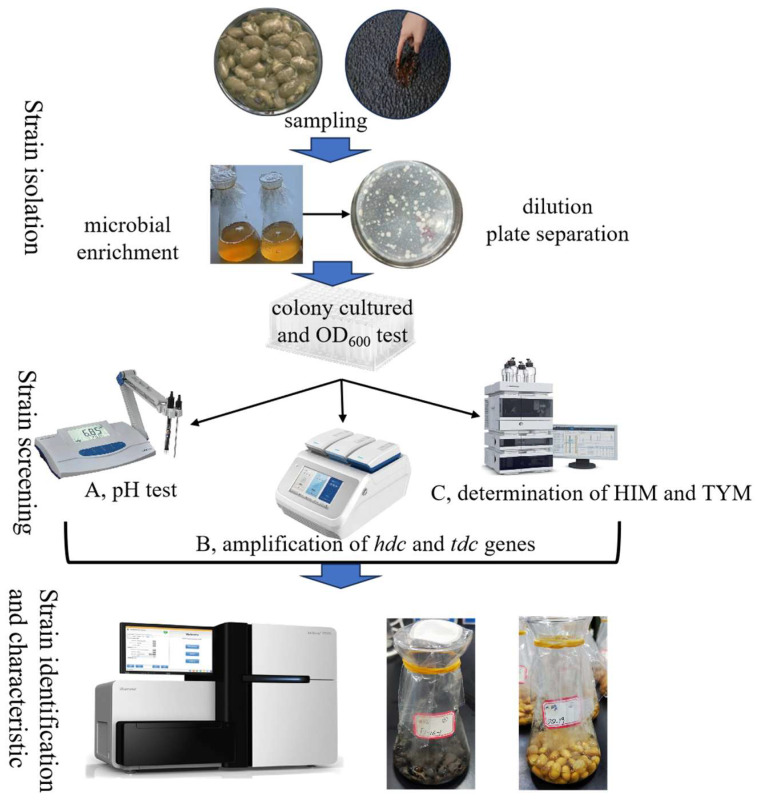
Diagram of the experimental design of this study.

During the process of fermenting soy sauce, the microbial community is highly diverse, which makes it difficult to identify BAs-producing strains. In this study, we utilized three distinct approaches to screen for the HIM- and TYM-producing strains that contribute the main BAs in soy sauce: (1) pH-based screening, (2) PCR amplification of the *hdc* and *tdc* genes, and (3) HPLC quantification of HIM and TYM content. A total of 118 isolates from *moromi* samples were purified and then analyzed using these methods (Figure 1).

**Figure 2:**
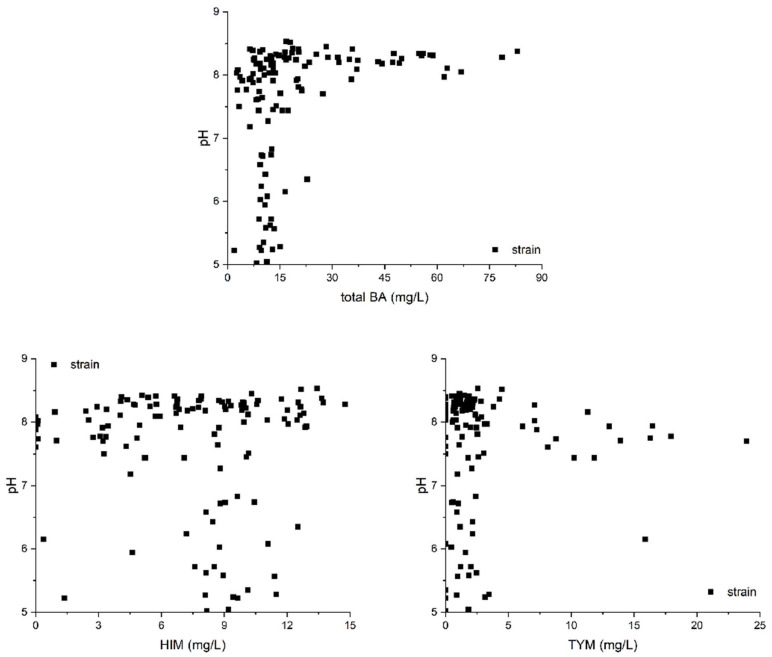
Correlation between pH and BA formation of isolated strains from fermented *moromi*.

The principle of pH-based screening relies on alkaline BA production, which leads to an increase in the pH of the strain culture medium [33]. Most previous studies utilized the principle of pH variation to screen for BAs-producing strains using a medium containing bromocresol purple indicator [34,35]. This approach is commonly adopted in the assessment of strains’ ability to produce BAs. Therefore, we analyzed the BAs content and pH of the supernatant of the cultured strain using HPLC and a pH meter. However, our results indicated no significant correlation between pH and HIM or TYM levels among the isolated strains (Figure 2). While only 19.49% of the tested strains exhibited both a high pH and high total BAs content, a considerable proportion of the strains produced increased BAs levels despite maintaining a low pH medium. This discrepancy can be ascribed to the simultaneous production of some acidic metabolites, such as lactic acid, by some BAs-producing strains, which counteracts the pH increase caused by BAs synthesis [36]. Additionally, we found that some isolates with a high pH medium produced a small amount of BAs, while some isolates with a low pH demonstrated significant HIM production; meanwhile, some isolates with a high pH produced low TYM content. These findings suggest that pH-based screening, although useful for preliminary identification, may lead to false positives and is not reliable for the definitive characterization of strains.

**Figure 3:**
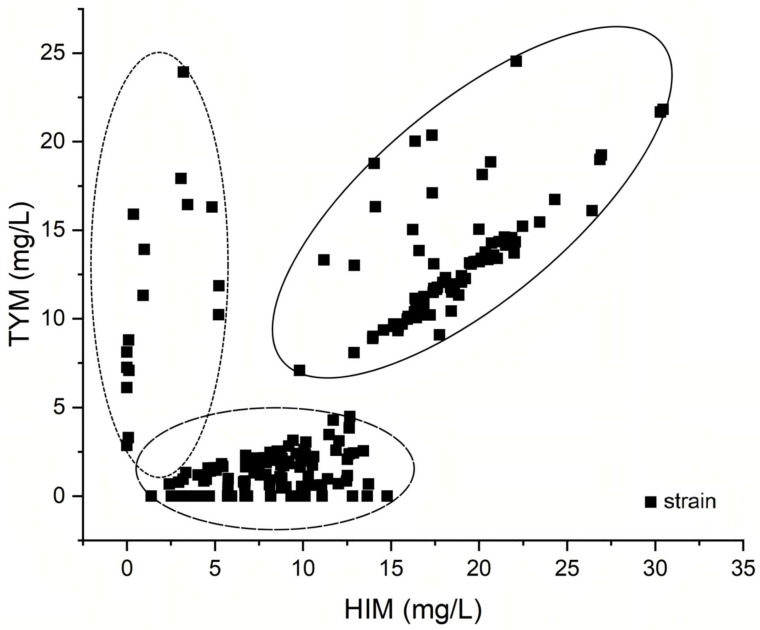
Production of HIM and TYM from all isolates from fermented *moromi*.

Upon analyzing the correlation between HIM and TYM generated by the strains isolated from *moromi* (Figure 3), an interesting finding emerged: 13.56% of strains demonstrate an extremely limited propensity for HIM generation. Half of the tested isolates are located in the bottom part of the figure, suggesting that they possess only a modest ability to form TYM. Additionally, approximately one-third of isolates were the target strains, which could generate both HIM and TYM (in an essentially linear relation, similar to the pattern detected in a previous study [9]). These results indicate that a significant portion of the isolates from *moromi* might generate both HIM- and TYM-decarboxylase, which can act on both histidine and tyrosine, as well as produce HIM and TYM.

**Figure 4:**
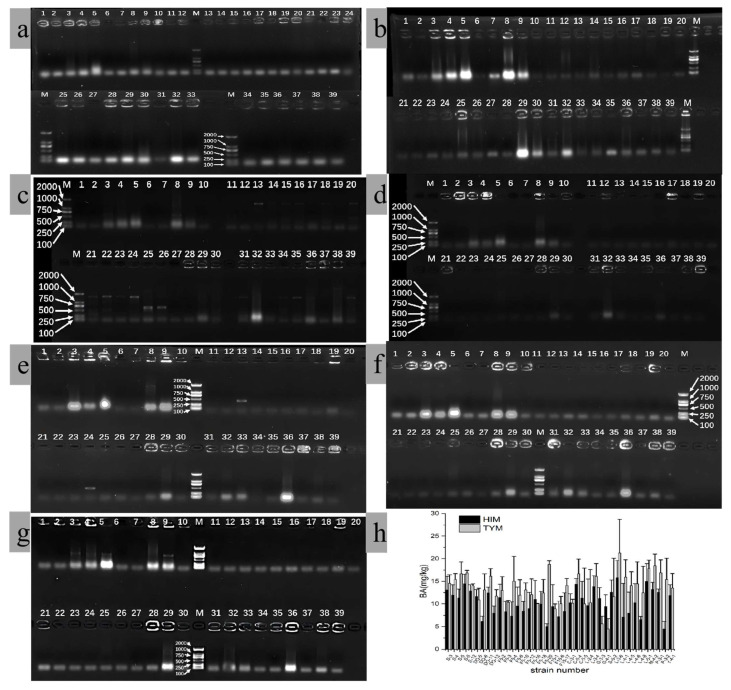
Results of *hdc* and *tdc* gene amplification in re-screened strains from fermented *moromi* ((**a**–**d**) represent *hdc* genes amplified using the primers of HDC-f/r, JV16HC/JV17HC, HDC3/HDC4, and HIS2-f/r, respectively; and (**e**–**g**) represent *tdc* genes amplified using the primers of TD5/TD2, TDC-P1/P2, and TDC-41/42, respectively; (**h**) refers to the formation of HIM and TYM in re-screened strains).

Additionally, we examined the *hdc* and *tdc* genes of 39 isolates that can simultaneously produce HIM and TYM by conducting an HPLC analysis, and the results are shown in Figure 4. The primers were derived from previous studies [30,31,32], and only 11 isolates presented the target band, indicating the presence of *hdc* or *tdc* genes. Among them, the HDC3/HDC4 primer set successfully amplified a 435 bp PCR product for strains L-2-4 (No. 25) and L-3-4 (No. 26). Strains S-5-2 (No. 29), L-4-5 (No. 32), and M-4-2 (No. 36) exhibited a distinct and clear PCR band of the expected size (531 bp) when using the HIS2-f/r HDC-specific primer. The TD5/TD2 TDC-specific primer set generated a typical 213 bp PCR product for strains F5-6 (No. 13) and C-5-5 (No. 24). Strains S-5 (No. 3), S-6 (No. 4), S-12 (No. 5), and DQ-12 (No. 9) displayed a unique and clear PCR band of the expected size (924 bp) when using the TDC-41/TDC-42 TDC-specific primer. However, the corresponding PCR bands were not detected in the other samples. The abortion of amplification may be attributed to variations in the gene clusters among different strains or the low homology of the decarboxylase genes across species [31,37]. This highlights a significant limitation of using universal primers for screening BAs-producing strains, as they may not bind to all variants of the target genes.

These results suggest that there is a weak correlation between the production of BAs and increasing the pH of the strain’s medium. The absence of *hdc* or *tdc* amplification does not indicate the inability of a strain to produce BAs; instead, it may reflect the limitations of the current primer design or the existence of alternative decarboxylation pathways. These findings underscore the importance of combining multiple screening methods to accurately identify BAs-producing strains.

### 3.2. Identification of HIM- and TYM-Producing Strains

**Figure 5:**
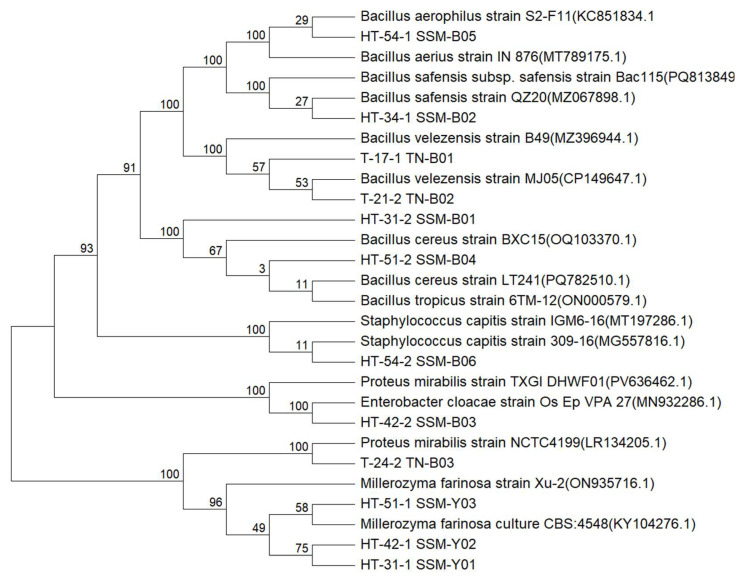
Phylogenic tree of isolated strains from fermented *moromi* (represented by a HT-prefixed identifier) and thua nao (represented by a T-prefixed identifier).

After the secondary screening using *moromi* and soybean media, nine isolates capable of producing both HIM and TYM were isolated from *moromi*, and three isolates that produce TYM were obtained from thua nao. Phylogenetic trees of the isolates based on 16S rRNA sequences and ITS rDNA genes were constructed (Figure 5). The nine isolates from *moromi* comprised four *Bacillus* strains, three *Millerozyma* strains, one *Enterobacter* strain and one *Staphylococcus* strain. The three isolates from thua nao included two *Bacillus* strains and one *Proteus* strain. The *Bacillus* that produces HIM and TYM was identified most frequently, but the species of *Bacillus* were diverse. Those that were most frequently reported were *Bacillus amyloliquefaciens* and *Bacillus velezensis*; for example, *Bacillus amyloliquefaciens* and *Bacillus* spp. isolated from *Cheonggukjang* produced 297.87 mg/L HIM and 123.08 mg/L TYM, respectively [38,39]. However, the production capability of BAs by *Bacillus altitudinis*, *Bacillus safensis* and *Bacillus tropicus* identified in this study has scarcely been reported. *Staphylococcus* is a common BAs-producing bacteria found in fermented soybean and sausage [40], and *Staphylococcus capitis* isolated from *Douchi* [41] produced the highest levels of HIM and TYM. For the first time, *Millerozyma farinosa* was isolated and shown to be able to produce both HIM and TYM, though it has been reported to have high BAs degradation rates in soy sauce [35]. This further explains that the generation of BA is a strain-dependent rather than genera trait. Although the genera of *Enterococcus* have frequently been reported to be able to produce HIM and TYM in animal-derived foods, such as *Enterococcus faecalis* isolated from traditional Spanish sausages [42] and *Proteus mirabilis* isolated from canned tuna [43], this is the first instance of these isolates being founded in fermented soybean foods.

### 3.3. HIM- and TYM-Forming Abilities of Screened Strains

The HIM and TYM production abilities of the screened strains are shown in Table 2. Among these strains, *Bacillus cereus*-HT-31-2 exhibited the highest capacity to produce both HIM and TYM, with values of 12.84 ± 1.90 mg/kg of TYM and 22.10 ± 0.47 mg/kg of HIM cultured in an amino acid medium. Normally, the release of proteases that thrive on soybean surfaces leads to the hydrolysis of soybean proteins into amino acids [44], and subsequent decarboxylation released by *Bacillus* species converts some of those amino acids into corresponding BAs [38].

Three strains of *Millerozyma farinose* isolated from *moromi* exhibited different abilities in the simultaneous production of HIM and TYM. *Millerozyma farinose*-HT-42-1 produced significantly more HIM in the amino acid medium, while the capacity for TYM production among the three strains remained similar. The *Enterobacter cloacae*-HT-42-2 and *Staphylococcus capitis*-HT-54-2 isolated from *moromi* showed a relatively weak ability to produce HIM and TYM simultaneously.

Our previous study showed that the thua nao products were contaminated with high levels of TYM, but TYM was rare [10]. In this study, the strains isolated from thua nao were shown to produce TYM, yet no HIM was detected. *Proteus mirabilis*-T-24-2 produced the highest TYM content, with a value of 22.66 ± 4.87 mg/kg that exceeded all *moromi* isolates in an amino acid medium. The other two screened strains, *Bacillus velezensis*-T-17-1 and *Bacillus velezensis*-T-21-2, produced TYM at levels exceeding 15 mg/kg, which were higher than those strains isolated from *moromi*.

Furthermore, *Bacillus tropicus*-HT-31-2 and *Millerozyma farinose*-HT-42-1 isolated from *moromi*, as well as three isolates from thua nao, were tested for their HIM and TYM formation capacity in a simulated medium made from *moromi* and boiled soybean. The results showed that *Bacillus cereus*-HT-31-2 cultured in a simulated medium produced higher contents of HIM and TYM than that cultured in the amino acid medium. However, the opposite result was found for *Millerozyma farinose*-HT-42-1. These results suggest that some *Bacillus* strains might be the crucial BAs producers in *moromi*. *Proteus mirabilis*-T-24-2 demonstrated a superior capacity for TYM production in the simulated soybean medium, achieving a production level of 160 mg/kg, while two *Bacillus* strains produced approximately 40 mg/kg TYM. These results indicate that different cultivation conditions have a significant impact on the production of HIM and TYM.

### 3.4. Characteristics of HIM and TYM Formation

**Figure 6:**
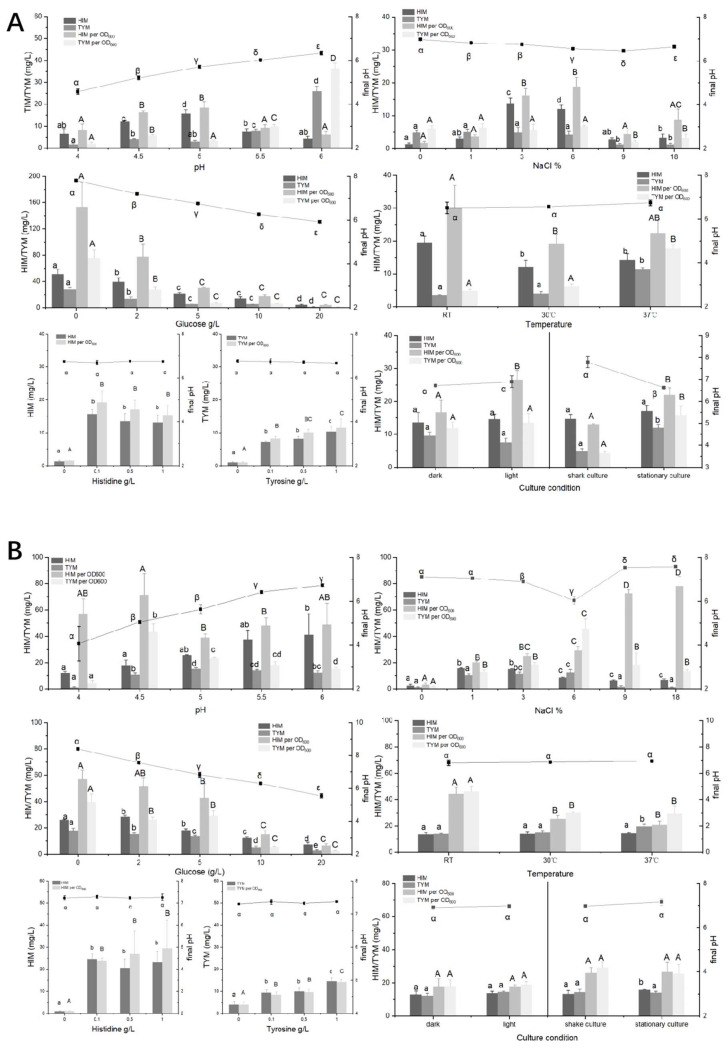
Effect of the culture environment on HIM and TYM formation in *Millerozyma farinose*-HT-42-1 (**A**) and *Bacillus cereus*-HT-31-2 (**B**) isolated from *moromi.* (Different symbol (Greek letters/capital English letters/lowercase English letters) indicate significant differences at the *p* < 0.05 level.)

**Figure 7:**
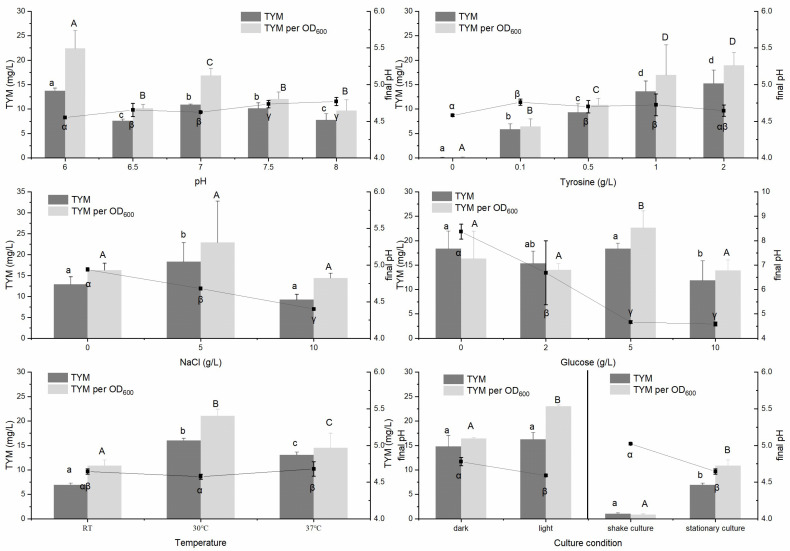
Effect of the culture environment on TYM formation in *Proteus mirabilis*-T-24-2 isolated from fresh thua nao. (TYM refers to TYM content, and TYM per OD600 indicates the amount of TYM that each unit of the strain can produce, Different symbol (Greek letters/capital English letters/lowercase English letters) indicate significant differences at the *p* < 0.05 level.)).

To determine the most important factors for HIM and TYM formation in fermented soybeans, the characteristics of potential HIM and TYM producer strains were evaluated. The results are depicted in Figure 6 and Figure 7.

For *Millerozyma farinose*-HT-42-1, moderate salinity (3–6%, *w*/*v*) was conducive to increasing the content of HIM and TYM, and a significant decline in HIM and TYM production was observed when salt concentrations were higher than 9%. A similar phenomenon occurred in *Millerozyma farinose*-HT-42-1 cultured in different pH media; the difference comprised a significant increase in TYM production, which was observed when the pH reached 6, indicating that HIM and TYM might not be associated with production. The initial fermentation stage of *moromi* typically begins at pH 6.2. Rapidly reducing the pH to approximately 5.5 during this stage can effectively reduce tyramine content. Interestingly, the increase in glucose in the medium inhibited the formation of HIM and TYM; both HIM and TYM remained at low levels when glucose levels exceeded 5 g/L. The formation of HIM and TYM changed very little when adding certain amounts of histidine and tyrosine, indicating that the method of reducing precursor amino acids might be ineffective. Moreover, culturing *Millerozyma farinose*-HT-42-1 at 37 °C with light in a stationary state allowed it to accumulate HIM and TYM. For *Bacillus cereus*-HT-31-2, the ability to form HIM and TYM under different conditions was higher than that of *Millerozyma farinose*-HT-42-1. HIM production showed a positive correlation with the increase in pH levels, while TYM production remained relatively stable. The production of HIM and TYM in response to varying concentrations of salinity, glucose, histidine, and tyrosine displayed a trend analogous to that observed in *Millerozyma farinose*-HT-42-1. Temperature and culture conditions had little effect on the amounts of HIM and TYM formed by *Bacillus cereus*-HT-31-2. Previous studies showed that a pH higher than 5 is favorable for the sensory properties of soy sauce [45]; thus, maintaining the *moromi* within a pH range of 5.0 to 5.5, along with high concentrations of glucose, might be beneficial for minimizing the production of HIM and TYM.

For *Proteus mirabilis*-T-24-2 isolated from thua nao, TYM formation was significantly reduced in alkaline environments compared to neutral and slightly acidic conditions. Additionally, the content of glucose and salt had only a limited impact on the production of TYM by *Proteus mirabilis*-T-24-2. Culturing *Proteus mirabilis*-T-24-2 at 30 °C with light under stationary conditions allowed it to form TYM.

Previous studies indicate that pH is a key factor influencing the activity of amino acid decarboxylase for the generation of BAs [46,47]. In this study, different pH values presented diverse effects on HIM and TYM production when using these three tested isolates. Moreover, it is not true that the higher the content of formed BAs (HIM and/or TYM), the higher the pH of the medium. BAs are widespread in salty fermented foods [6,18,19], and the BAs production abilities exhibited by different strains under the influence of salt were diverse. The production of HIM by two isolates from *moromi* was higher than that of TYM in a high concentration of NaCl. The HIM per OD600 of *Bacillus cereus*-HT-31-2 increased with the increase in the NaCl concentration, and the NaCl concentration had little effect on the formation of TYM by isolates from thua nao. Similarly, the increase in glucose levels inhibited HIM and TYM production for two isolates from *moromi* but exerted a minimal effect on *Proteus mirabilis*-T-24-2 from thua nao. Interestingly, the final pH of the medium in which the three isolates were cultivated decreased with the addition of glucose compared to the initial pH of the medium; moreover, the higher the glucose content, the lower the final pH became. The culture conditions, such as light, temperature, and aeration, exerted little influence on the production of HIM and TYM but had an impact on the growth of the strain.

## 4. Conclusions

In this study, we conducted a comparison and presented a detailed analysis of pH screening and target gene amplification methods for identifying crucial HIM- and TYM-producing strains from fermented soybean foods. We demonstrated that they were inappropriate compared with HPLC determination due to the high rate of false positive results. Two isolates, *Bacillus cereus*-HT-31-2 and *Millerozyma farinosa*-HT-42-1, were screened in *moromi* and shown to have a strong ability to produce HIM and TYM. Additionally, an isolate, *Proteus mirabilis*-T-24-2, was screened in thua nao and identified as a key strain for TYM formation. The characteristics of HIM and TYM formation by these three isolates were tested and yielded different results. The constituents of the medium, such as the pH, NaCl, and glucose concentrations, had a significant effect on the formation of HIM and TYM, but different strains exhibited considerable differences. The culture conditions were shown to exert little influence on HIM and TYM formation. This study provides valuable data and a theoretical foundation for understanding BAs-producing strains, and it will be beneficial for further control of HIM and TYM in fermented soybean foods.

## Figures and Tables

**Table 1:** The primers used in this study.

Target Gene	Primer	Sequence (5′-3′)	Amplicon Size (bp)	Note	Reference
*hdc*	JV16HC	AGATGGTATTGTTTCTTATG	367	G+	[30]
	JV17HC	AGACCATACACCATAACCTT			
	HDC-f	TCHATYARYAACTGYGGTGACTGGRG	709	G−	
	HDC-r	CCCACAKCATBARWGGDGTRTGRCC			
	HDC3	GATGGTATTGTTTCKTATGA	435	G+	[31]
	HDC4	CAAACACCAGCATCTTC			
	HIS2-f	AAYTSNTTYGAYTTYGARAARGARGT	531	G−	
	HIS2-r	TANGGNSANCCDATCATYTTRTGNCC			
*tdc*	TDC-P1	CCRTARTCNGGNATAGCRAARTCNGTRTG	1100		
	TDC-P2	GAYATNATNGGNATNGGNYTNGAYCARG			
	TDC-41	CAYGTNGAYGCNGCNTAYGGNGG	924		[32]
	TDC-42	AYRTANCCCATYTTRTGNGGRTC			
	TD5	CAAATGGAAGAAGAAGTAGG	213		
	TD2	ACATAGTCAACCATRTTGAA			

Note: Y: T/C, N: A/T/C/G, R: A/G, S: G/C, W: A/T, D: G/A/T, K: G/T, B: G/T/C, H: A/T/C; G+: Gram-positive bacteria; G−: Gram-negative bacteria.

**Table 2:** HIM and TYM formation capacity of screened strains.

Strain		Amino Acid Medium		*Moromi* or Soybean Medium	
		HIM mg/kg	TYM mg/kg	HIM mg/kg	TYM mg/kg
*Moromi*	*Millerozyma farinosa*-HT-31-1	4.89 ± 0.35	9.78 ± 1.45		
	*Bacillus cereus*-HT-31-2	22.10 ± 0.47	12.84 ± 1.90	43.62 ± 3.46	26.03 ± 3.13
	*Bacillus safensis*-HT-34-1	17.56 ± 3.41	11.81 ± 1.83		
	*Millerozyma farinosa*-HT-42-1	26.52 ± 2.42	11.15 ± 1.35	10.21 ± 1.73	8.27 ± 0.83
	*Enterobacter cloacae*-HT-42-2	4.30 ± 1.19	8.14 ± 1.49		
	*Millerozyma farinose*-HT-51-1	5.79 ± 0.75	11.54 ± 0.79		
	*Bacillus cereus*-HT-51-2	4.35 ± 1.45	8.43 ± 0.53		
	*Bacillus aerophilus*-HT-54-1	13.41 ± 1.08	10.61 ± 1.04		
	*Staphylococcus capitis*-HT-54-2	4.89 ± 0.74	8.96 ± 0.89		
Thua nao	*Proteus mirabilis*-T-24-2		22.66 ± 4.87		159.55 ± 6.56
	*Bacillus velezensis*-T-17-1		17.34 ± 0.42		40.87 ± 5.27
	*Bacillus velezensis*-T-21-2		16.44 ± 2.14		40.36 ± 10.93

Note: *B. tropicus*-HT-31-2 showed significantly higher HIM production than other *Bacillus* spp.

## Data Availability

The original data presented in the study are included in the article, and further inquiries can be directed to the corresponding authors.
